# Cardiac MRI in MINOCA: Current Evidence, Parametric Mapping Advances, and Future AI Applications—A Systematic Review

**DOI:** 10.3390/diagnostics16091307

**Published:** 2026-04-27

**Authors:** Diana Alexandra Pepelea, Roxana E. Coroiu, Eliza M. Aron, Ramona M. Popa, Mircea D. Hogea, Rosana M. Manea

**Affiliations:** 1 Clinical Laboratory of Radiology and Medical Imaging, Brasov’s Emergency County Hospital, Calea Bucuresti, Nr. 25-27, 500326 Brasov, Romania; 2 Faculty of Medicine, Transilvania University, Str. Nicolae Balcescu, Nr. 56, 500019 Brasov, Romania

**Keywords:** MINOCA, myocardial infarction with nonobstructive coronary arteries, cardiac magnetic resonance, T1/T2 mapping, extracellular volume (ECV), late gadolinium enhancement (LGE), diagnostic yield, Radiomics/AI

## Abstract

**Background**: Myocardial infarction with nonobstructive coronary arteries (MINOCA) is a heterogenous clinical syndrome in which aetiologies range from “true” ischemic mechanisms to non-ischemic mimics (e.g., myocarditis and Takotsubo syndrome). Cardiac magnetic resonance (CMR) plays a central role in the diagnostic pathway. Recent advances in parametric mapping (native T1, T2, and extracellular volume ECV) and evolving AI/radiomic methods promise to further improve diagnostic accuracy and prognostic stratification. This review aims to evaluate the current CMR evidence in MINOCA, while highlighting parametric mapping advances and future directions in the sphere of AI and radiomics. **Methods**: A systematic literature search of PubMed and the Directory of Open Access Journals (DOAJ) was performed. We included original prospective and retrospective CMR studies of MINOCA and MINOCA-like presentations in adults. Data were extracted into a master dataset and synthetised thematically into five subsections: (1) diagnostic yield, (2) reclassification rate), (3) timing of CMR, (4) prognosis, and (5) future directions. **Results**: Twenty-two studies met the inclusion criteria. CMR diagnostic yield varied by protocol and timing but was consistently substantial. CMR consistently reclassified initial MINOCA diagnoses (ischemia or alternative non-ischemic diagnoses). Parametric mapping provided incremental diagnostic and prognostic information. Across studies, early imaging (ideally within the first 1–2 weeks) increased diagnostic yield, while delayed CMR reduced detectability of transient lesions. Early AI and radiomics work show promise for LGE-based classification and for predicting post-contrast findings from non-contrast data, but current models require larger, multicentre training and robust external validation. **Conclusions**: CMR increases diagnostic yield and reclassification rates in MINOCA, particularly when performed early and with standardised T1/T2/ECV mapping. Mapping not only improves detection of inflammatory and diffuse injuries but also contributes to prognostic stratification. High-resolution LGE, OCT, and AI/radiomics are promising future refinements but need prospective validation in large, early, mapping-inclusive cohorts.

## 1. Introduction

Myocardial infarction with nonobstructive coronary arteries (MINOCA) is defined as the presence of acute myocardial infarction (AMI) according to the fourth universal definition of MI [1], occurring in the absence of obstructive coronary artery disease (CAD) on invasive coronary angiography (<50% stenosis). It accounts for roughly 5–10% of cases presenting with acute coronary syndrome [2].

MINOCA aetiologies are heterogenous, ranging from “true” ischemic causes (e.g., plaque disruption, coronary spasm, thromboembolism, spontaneous coronary artery dissection, and microvascular obstruction), to non-ischemic entities, also known as MINOCA mimics (e.g., myocarditis, Takotsubo syndrome, and cardiomyopathies). Differentiating acute ischemic injury from non-ischemic conditions remains challenging in clinical practice. As such, the term MINOCA is often used as a workup diagnosis until further examinations either confirm its ischemic nature or present an alternative cause [2,3].

Given this diversity, cardiac magnetic resonance (CMR) has emerged as a pivotal non-invasive imaging modality in the evaluation of MINOCA patients by combining functional assessment with tissue characterisation to help distinguish ischemic injury from inflammation or other cardiomyopathies. Conventional CMR components (cine imaging, T2-weighted sequences, and late gadolinium enhancement-LGE) allow detection of wall-motion abnormalities, oedema, and focal necrosis or fibrosis, which substantially increase diagnostic yield and often lead to reclassification of an initial MINOCA label into a more specific diagnosis (infarction, myocarditis, Takotsubo syndrome, etc.) [3,4].

Recent advances in CMR include multiparametric mapping sequences that further extend the diagnostic capabilities of CMR [5] by allowing a quantitative measure of native T1 and T2 relaxation times and extracellular volume (ECV), which can directly assess the presence of oedema, fibrosis, or diffuse myocardial injury. Consensus recommendations [5] outline their technical basis, clinical indications, and acquisition standards for T1/T2/ECV mapping and emphasise their utility in acute myocardial disease, where conventional weighted sequences may be insensitive. Consequently, parametric mapping has rapidly become an important component of modern CMR protocols for acute myocardial injury and is particularly valuable in MINOCA, where diffuse or transient tissue changes are common and qualitative techniques can sometimes be insufficient.

Artificial intelligence (AI) and radiomics are emerging techniques that are transforming many aspects of cardiac magnetic resonance imaging [3]. Early studies [6,7] report promising results both at acquisition levels (automation allows higher spatial/temporal resolution, reduces artifacts with motion-correction), as well as in the post-processing stage (segmentation of cardiac chambers and myocardium, volumetric outputs, and prediction of post-contrast information from non-contrast images), features which could ultimately allow for faster, shorter protocols, and help prioritise which patients require contrast. The diagnostic challenges presented by MINOCA make it ideally suited for AI and machine learning techniques, as some early studies have sought to validate the use of AI models in discriminating ischemia from inflammatory enhancement (useful in the common myocardial infarction vs. myocarditis dilemma) [6,8], with promising results. As it stands, before wide-range clinical use in MINOCA, AI models must be trained and validated on large multicentric cohorts and be integrated into imaging workflows to demonstrate their diagnostic and triage value.

The primary aim of this study was to evaluate the role of cardiac MRI in the diagnosis of MINOCA, with special emphasis on parametric mapping techniques—including T1, T2, and ECV (extracellular volume). A secondary aim was to highlight the emerging role of advanced techniques and artificial intelligence (AI) applications in this context.

## 2. Materials and Methods

**Figure 1:**
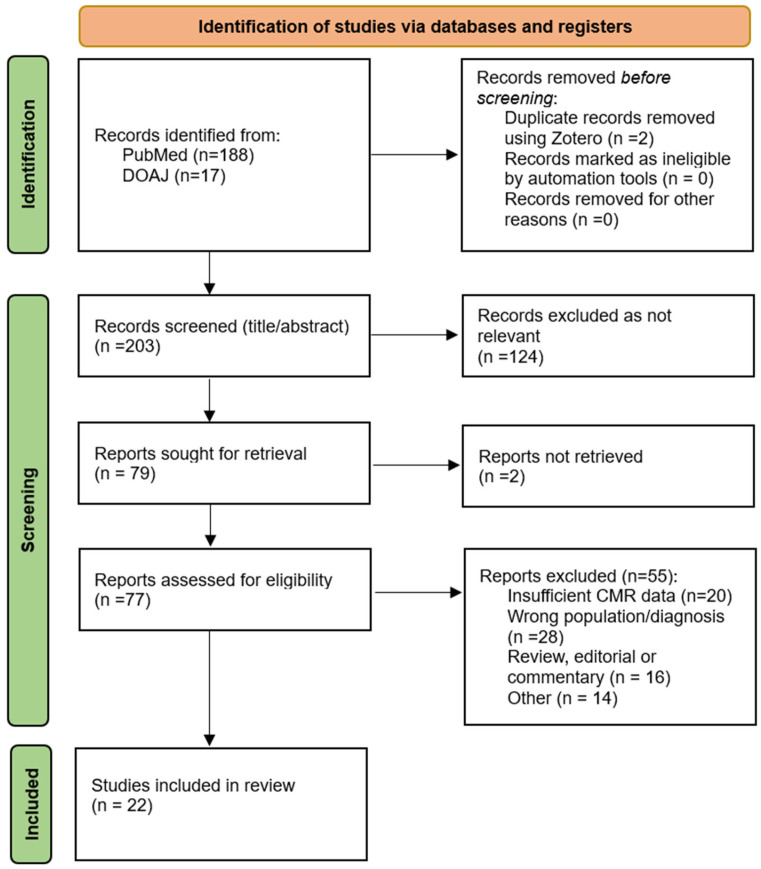
PRISMA flow diagram of study selection. Records were identified via PubMed (*n* = 188) and DOAJ (*n* = 17). After removal of duplicates (*n* = 2), 203 unique records were screened by title and abstract. Of these, 124 were excluded as not relevant, and 79 were sought for retrieval. Seventy-seven full-text articles were assessed for eligibility, among which 55 were excluded for reasons detailed in the Methods (e.g., wrong population/diagnosis, insufficient CMR data, review/editorial, other). Twenty-two studies were included in the qualitative synthesis.

This literature review was conducted as a systematic review of published studies examining cardiac magnetic resonance (CMR) in patients with myocardial infarction with non-obstructive arteries (MINOCA), with particular emphasis on advanced parametric mapping techniques and emerging artificial intelligence (AI) applications. The review was performed in accordance with the Preferred Reporting Items for Systematic Reviews and Meta-analyses (PRISMA) guidelines, and the study selection process is illustrated in the PRISMA flowchart in Figure 1.

A systematic literature search was performed in the PubMed and Directory of Open Access Journals (DOAJ) databases. The search strategy combined controlled vocabulary (MeSH) and free-text keywords for MINOCA (e.g., “MINOCA,” “myocardial-infarction with non-obstructive coronary arteries,” and “non-obstructive ACS”), cardiac MRI (e.g., “cardiac MRI,” “CMR,” and “cardiac magnetic resonance”) and parametric mapping (e.g., “T1 mapping,” “T2 mapping,” “ECV,” and “extracellular volume”). Boolean operators were used to refine the search and ensure a comprehensive review of the relevant literature.

The inclusion criteria were as follows: original research (prospective or retrospective observational studies); adult populations (≥18) presenting with a working diagnosis of MINOCA (presenting with MINOCA or acute myocardial injury syndromes clinically compatible with MINOCA—e.g., myocarditis and Takotsubo syndrome); cardiac MRI performed in the diagnostic work-up; studies reporting mapping or LGE/T2WI data (mapping data will be extracted whenever present); studies reporting at least one of the following: diagnostic yield, reclassification rate, prognostic outcome, and date regarding the appropriate timing of CMR; published 2015–present. Paediatric studies, case reports or small series, reviews, meta-analyses or editorials, studies not involving cardiac MRI in the diagnostic evaluation, studies with insufficient CMR imaging data relevant to the outcomes of interest, as well as non-English publications were excluded.

Duplicate records were removed using Zotero reference management software (version 8.0.4) prior to screening. Two reviewers (PAD and AEM) independently screened the remaining titles and abstracts to assess their relevance to the research question. Studies that were considered potentially eligible were retrieved in full text and evaluated against the predefined inclusion and exclusion criteria. Any disagreements in study selection were resolved by consensus and discussion by a third reviewer (CER).

Risk of bias in the included studies was assessed using the QUADAS-2 tool, which evaluates four domains: patient selection, index test, reference standard, and flow and timing. The tool was tailored to the review question to account for the use of CMR as the index test in patients with suspected MINOCA. Each study was evaluated as having low, high, or unclear risk of bias in each of these domains, as well as concerns regarding applicability where appropriate

The primary outcome of this study was to assess the diagnostic yield of cardiac MRI, with a focus on parametric mapping, by way of imaging endpoints (LGE presence, pattern of oedema, and quantitative mapping values) on the diagnosis of patients with MINOCA.

A secondary outcome of this study was to evaluate the contribution of cardiac MRI in the diagnostic and reclassification rate of initial MINOCA diagnosis and MINOCA-mimickers (e.g., myocarditis and Takotsubo syndrome).

Additional objectives of this article were to assess the prognostic implications of cardiac MRI in MINOCA, as well as explore how the timing of CMR influences diagnostic performance and outcomes.

Finally, the possible applications of advanced imaging techniques, as well as artificial intelligence (AI) and radiomics, relevant in the context of MINOCA were also analysed.

## 3. Results

**Figure 2:**
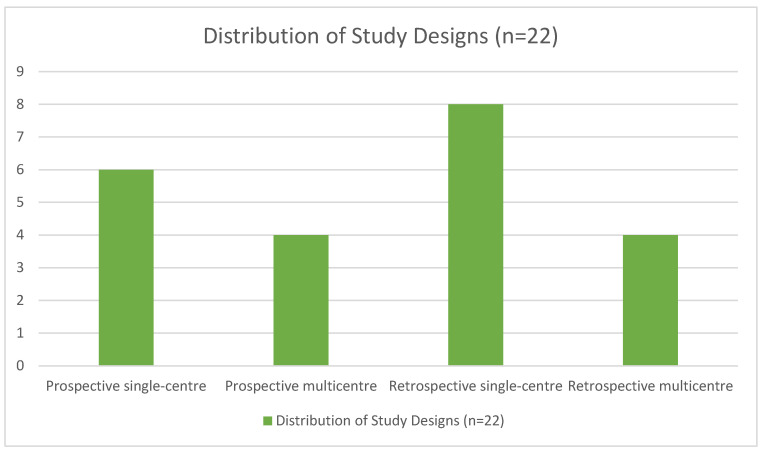
Distribution of study designs.

A total of 22 articles published between 2015 and 2025 were analysed. The studies included patients who underwent CMR for MINOCA diagnosis or to help distinguish it from other similar, non-ischemic conditions (such as myocarditis or Takotsubo syndrome). The number of patients included in the studies ranged from *n* = 38 to *n* = 1596. As shown in Figure 2, among the 22 articles included, 6 were prospective single-centre, 4 were prospective multicentre, 8 were retrospective single-centre, and 4 were retrospective multicentre. All studies included a conventional CMR protocol (adapted to each specific institution), while five of them also included T1/T2 mapping sequences.

Methodological quality assessment using the QUADAS-2 tool showed that the overall risk of bias varied across studies. The most frequent concerns were related to the patient selection and flow-timing, whereas the index test domain was generally judged to be at lower risk when CMR protocols were clearly described. Overall, QUADAS-2 suggested that the evidence so far is heterogenous, with the timing of CRM remaining a key source of bias across studies, highlighting the need for more standardised diagnostic workflows in MINOCA.

To help exemplify the role of CMR in MINOCA diagnosis workup, we have organised the results into five thematic subsections: (1) diagnostic yield, (2) reclassification rate, (3) timing of CMR, (4) prognosis, and (5) future directions (advanced techniques and complementary examinations such as HR-LGE, OCT, and the role of AI). Each included study was reviewed for the themes it addressed, and is therefore reported in every relevant subsection. To emphasise this, we present a concise table (Table 1) listing the key design features. Furthermore, as this review emphasises the role of parametric mapping and advanced techniques, we also include a table subcategory to characterise this subgroup.

### 3.1. Diagnostic Yield

Cardiac magnetic resonance increases diagnostic yield in MINOCA, as demonstrated across a range of studies. Reported CMR diagnostic yields vary by timing, protocol, and reader experience but are higher than clinical or angiographic assessment alone—for example, early, mapping-inclusive CMR raised the diagnostic yield from 47% to 77% in the SMINC program [11] (SMINC-1 vs. SMINC-2), and cohort scanning within 14 days with higher troponin report yielded up to 94% in optimally selected subgroups [14]. Systematic low-threshold use of CMR was associated with a near five-fold increase in myocarditis detection at the population level in a large single-centre implementation study [18], and pre-angiography CMR [15] localised an infarct-related artery or identified alternative non-ischemic pathology in a substantial minority (new IRA diagnosis in 23%; non-ischemic pathology in 14%), illustrating CMR’s reclassification power.

In the prospective SMINC-2 cohort [11] (*n* = 148), early comprehensive CMR performed at a median of 3 days after admission returned a diagnosis in 77% of MINOCA patients, compared with 47% in the historic SMINC-1 cohort [29] imaged at a median 12 days. The increase in diagnostic yield reflected larger proportions of myocarditis (17% vs. 7%) and Takotsubo syndrome (35% vs. 19%); the proportion classified as myocardial infarction was similar between studies (22% vs. 19%). Native T1 mapping and ECV were integral to SMINC-2’s protocol and were used to define oedema and diffuse injury. Compared with SMINC-1, the numbers of patients with wall-motion abnormalities, oedema, and LGE were higher in SMINC-2, and the rate of “undecided/indeterminate” studies fell to near zero.

In a multicentre comparison of a short non-contrast CMR protocol ShtCMR (cine, T2WI, and native T1 and T2 mapping) versus a standard protocol StdCMR, including LGE and post-contrast T1 mapping, Gatti et al. [10] demonstrated that mapping-based CMR achieved identical diagnoses in 85% of MINOCA patients when interpreted by an expert reader. Diagnostic agreement increased to 99% when expert readers reported high diagnostic confidence, whereas agreement was lower for non-experts (73% overall, rising to 89% with good confidence). For expert readers, the mapping-only protocol showed robust diagnostic performance (sensitivity and specificity 92%) and effectively distinguished myocarditis (87% agreement) and Takotsubo syndrome (83%). However, the absence of LGE significantly weakened the ability to detect myocardial infarction: agreement for MI was 77% in expert readers and only 43% in non-experts. Overall, the mapping-only protocol shortened scan time by more than half (21 ± 9 vs. 45 ± 11 min) but underestimated pathology extent and missed small infarcts that were visible only on LGE imaging.

Furthermore, another retrospective cohort [16] demonstrated that the use of CMR in patients with high troponin levels helped provide a diagnosis in 85% of cases, thus allowing better therapeutical strategies in accordance with specific aetiologies.

Several of the larger MINOCA cohorts [13,14,18,25] explicitly acknowledged the absence of standardised T1/T2/ECV mapping as a limitation and suggested that quantitative parametric techniques could have increased diagnostic sensitivity and improved case reclassification.

### 3.2. Reclassification Rate

CMR can further refine the diagnostic labelling of MINOCA patients, which has a direct effect on management, treatment strategies, and prognosis based on the cause (or at least more specific categorisation).

One study [12] examined the incremental value of high-resolution LGE(HR-LGE) in MINOCA patients. They observed that the addition of HR-LGE to a conventional CMR protocol increased the rate of definite myocardial LGE (64% vs. 51% with conventional LGE) and raised the proportion of patients with a definite final CMR diagnosis from 50% to 71%; HR-LGE changed the final diagnosis in 45/172 patients (26%) and reduced inconclusive studies from 50% to 29%.

In a prospective study [15] of 70 NSTEMI (non-ST-segment elevation myocardial infarction) patients who underwent CMR with LGE before coronary angiography (CAG), LGE-CMR identified an infarct-related artery (IRA) in 67.1% of patients compared with 61.4% by angiography; CAG failed to localise an IRA in 38.6% (27/70), among whom LGE-CMR localised the IRA in 48.1% (13/27), and revealed a non-ischemic diagnosis in 18.5% (5/27). Overall, LGE-CMR identified an IRA diagnosis in 23% (16/70) and a new non-ischemic diagnosis in 14% (10/70) of the cohort. Subgroup analysis showed that in patients with non-significant coronary disease on angiography (*n* = 26), LGE identified an IRA in 53.8% versus 19.2% for CAG. At the study level, 18.6% (13/70) had no LGE.

Another study [18] reported that low-threshold use of CMR in patients with MINOCA increased the identification of myocarditis, thus aiding with the reclassification process and leading to better management and treatment strategies. Of 556 total CMRs, myocarditis was diagnosed in 76 (13.7%) overall. The authors used T2-weighted imaging for oedema and LGE for necrosis; parametric T1/T2 mapping was not available in this dataset.

### 3.3. Prognostic

A number of studies showed that CMR-based diagnosis is associated with different prognoses and could provide incremental risk stratification beyond angiography and clinical features.

In larger cohorts, CMR diagnosis categories showed distinct outcomes. Dastidar et al. [20] (median CMR at 37 days) found that MINOCA patients that has sustained a myocardial infarction (ischemic aetiology) had increased mortality rates (4.5% over a median of 3.5 years), and that the cardiomyopathy/Takotsubo group had the highest mortality (15% vs. 4.5% MI vs. 2% myocarditis/normal). Long-term follow-up data [21] demonstrated that CMR-confirmed acute myocardial infarction (vs. myocarditis/Takotsubo) was strongly associated with later MACE (major adverse cardiovascular events)—24% overall MACE over a median of 7.1 years. Other studies reported similarly elevated event rates, as well as the impact of LGE extension: involvement of ≥2–3 segments increased MACE risk [22].

The study by Bergamaschi et al. [9] focused on the prognosis of true MINOCA (the cohort excluded non ischemic aetiologies) by classifying patients into three subgroups based on their CMR phenotypes: patients with both late gadolinium enhancement (LGE) and abnormal mapping (M) values (LGE+/M+), patients with regional ischemic injury with abnormal mapping but no LGE (LGE−/M+), and patients with normal CMR (LGE−/M−). By using a comprehensive protocol, including T1 and T2 mapping and quantified LGE, they demonstrated the existence of a high-risk subgroup with combined LGE and abnormal mapping (LGE+/M+), and that both %LGE and T2 mapping values were independent predictors of subsequent MACE.

Another retrospective cohort [26] demonstrated that among all included cases, only 7% were classified as true MINOCA, while 8% were MINOCA mimickers and 85% had an ischemic aetiology. Despite patients with true MINOCA being significantly fewer, as well as having fewer cardiovascular risk factors, lower biomarker levels, and more favourable echocardiographic characteristics, the overall long term all-cause mortality in patient with true MINOCA versus those with obstructive coronary artery disease was similar (32.1% vs. 30.9%).

Finally, in their study, Bucciarelli et al. [17] showed that the LGE pattern (especially transmural) could stratify risk and lead to treatment changes. On the same note, LGE transmural extent and ST-elevation predicted early ventricular arrhythmia risk during hospitalisation [23].

### 3.4. Timing

One study [14] prospectively studied 719 patients with suspected ACS (acute coronary syndrome) and nonobstructive coronaries who underwent CMR; the overall diagnostic yield was 74%. Among the 198 patients with a scan interval of <14 days and peak troponin of ≥211 ng/L, 186 (94%) had a diagnostic CMR study, versus 72% diagnostic yield when CMR was performed at ≥14 days. For patients with troponin <211 ng/L, the diagnostic yield was 76% if CMR was performed at <14 days but only 53% if scanned at ≥14 days. Multivariable analysis showed that a scan interval of <14 days and peak troponin of ≥211 ng/L were independent predictors of a diagnostic CMR. The authors reported a positive linear relationship between peak troponin and number of LGE and oedema segments, supporting the biologic plausibility that greater myocardial injury increases CMR detectability. The study did not include T1/T2 mapping sequences; the authors noted that mapping may have further increased sensitivity and specificity.

As mentioned before, the study by Sörensson [11] placed early CMR with parametric mapping at centre-stage, as it demonstrated that mapping helps detect oedema and transient conditions (e.g., myocarditis and Takotsubo syndrome) that are time-sensitive, thus consolidating its role in the work-up of MINOCA. As previously stated, the authors also acknowledged that the use of T1/T2 mapping could further increase sensitivity for more subtle or diffuse lesions. That being said, systematic early CMR (ideally within days) and ideally incorporating standardised parametric mapping as well as LGE could maximise the reclassification of MINOCA patients and minimise false negatives as a result of late imaging.

Another study [18] demonstrated that, other than the impact on the reclassification rate of MINOCA cases, CMR timing still matters: oedema and some LGE patterns are dynamic and can resolve, and the authors acknowledged that CMRs were performed up to 90 days from symptom onset, so late scanning may reduce detectability of transient findings.

The retrospective study by Macedo Conde et al. [13] evaluated the diagnostic performance of CMR in patients with an initial diagnosis of myocardial infarction with nonobstructive coronary arteries (MINOCA). A total of 163 patients who were assessed with CMR within 180 days of hospital admission were included. In this cohort (*n* = 163), CMR identified a cause in 74.2% of patients with suspected AMI and non-obstructive coronaries (MINOCA 31.3%, Takotsubo 22.7%, and myocarditis 20.2%); 25.8% had a normal/indeterminate CMR. Time-to-scan was strongly associated with yield: 50.9% (83/163) were scanned at ≤14 days, 16.6% (27/163) at 15–30 days, and 32.5% (53/163) at >30 days. Performing CMR within 14 days was associated with a significantly higher probability of obtaining a diagnosis (56.2% of diagnosed patients scanned at ≤14 days vs. 35.7% of undiagnosed). The authors concluded that CMR should ideally be performed within the first 14 days to maximise diagnostic yield (in this case, the diagnostic yield was as high as 94%). The authors also acknowledged that if parametric techniques were added, sensitivity would further increase. These findings align with the results of the studies mentioned before and justify the use of early CMR and conventional mapping protocols to improve the diagnostic yield.

Lastly, the study conducted by Dastidar et al. [20] performed CMR over a median of 37 days from presentation, with a diagnostic yield of 74%, which was lower than the other studies previously mentioned, and the authors argued that delayed timing reduced oedema detection, while earlier scanning (preferably within the first week of acute presentation) would improve detection.

### 3.5. Future Direction

The role of multimodality imaging by combining optical coherence tomography (OCT) and CMR in the diagnosis of MINOCA is another point of interest included in this review. Despite it being an underused technique, in one study [19], the authors utilised a combination of OCT and CMR to establish a diagnosis, with 84% accuracy (84/116 cases), as opposed to their separate use, where OCT identified a possible/definite lesion in just 46% of cases and CMR alone in 53% of cases. In the study by Gerbaud et al. [27], 40 patients with suspected epicardial MINOCA underwent both OCT and CMR imaging: CMR identified acute myocardial infarction in 31 patients (77.5%), while OCT identified the potential coronary lesions including plaque rupture (35%), plaque erosion (30%), thrombus (7.5%), coronary artery dissection (5%), and calcified nodules (2.5%). What is remarkable is that through the combined use of both OCT and CMR, the underlying mechanism and diagnosis were identified in 100% of cases. Similarly, Opolski et al. [28] evaluated 40 patients using OCT, while CMR was performed in 31 patients, and found that LGE was useful in identifying infarct-related myocardial territory and thus enabled correlation with coronary plaque morphology as detected by OCT. The authors concluded that multimodality imaging is complementary and markedly increases diagnostic yield.

In their study, Litingre et al. [12] (*n* = 229) provided strong evidence that improving LGE spatial resolution substantially reduces inconclusive CMRs and can both reveal tiny subendocardial infarcts and reclassify apparent non-ischemic patterns. The free-breathing HR-LGE approach reduced voxel volume roughly four times compared to conventional breath-held LGE, which translated into a 21% increase in definite diagnoses among the HR-imaged subgroup, changes that have clear therapeutic and prognostic implications.

Two studies on AI and machine learning (ML) addressed the problems of reclassification and contrast-sparing imaging that are encountered in MINOCA patients. In the retrospective series [6], 173 patients were analysed, and it was shown that radiomics applied to LGE regions can discriminate myocardial infarction from myocarditis with high accuracy; radiomics outperformed less-experienced readers and produced similar results to more expert readers. By contrast, efforts to predict post-contrast information from pre-contrast cine images (i.e., to identify the presence, location, and extent of scarring without gadolinium) have produced more modest but encouraging results, as shown in the study conducted by Abdulkareem et al. [7]. Such studies are small, single-centre, and have limited data, which shows the need for more future work to validate their use in MINOCA patients. In clinical practice, MINOCA is a working diagnosis; therefore, it needs further analysis to identify its ischemic or nonischaemic aetiology. Many studies validate the use of CMR in the workup of MINOCA; however, further proof is needed to assess the routine performance of more advanced and complementary imaging techniques, as those previously mentioned.

## 4. Discussion

The diagnosis in MINOCA patients can sometimes prove to be difficult, and misdiagnosis could lead to improper treatment and aggravation of disease. Therefore, it is essential to have a clear understanding of the imaging characteristics of MINOCA patients, both in terms of standard imaging practices as well as novel ones (such as parametric mapping and other advanced complementary techniques). Keeping this in mind, the advantages of CMR, as demonstrated in the aforementioned studies, are improved diagnostic yield and reclassification rate as well as the ability to derive prognostic data and guide management.

Firstly, as mentioned before, the causes of MINOCA are diverse, and identifying ischemic versus non-ischemic aetiologies is paramount to receiving proper treatment. The diagnosis yield of CMR is indisputable, as demonstrated in the studies included. The improvements in diagnostic yield can be explained by three complementary advances: (1) earlier scanning captures oedema or other transient lesions, (2) parametric mapping (native T1/T2/ECV) detects diffuse or subtle injury that T2-weighted imaging misses, and (3) higher spatial resolution (HR-LGE) and multimodality strategies (e.g., OCT + CMR) reveal small infarcts or intraluminal causes that angiography or CMR alone can miss.

Some studies [13,14,18,25] clearly noted that T1/T2/ECV mapping would have been a beneficial addition to their protocols to improve diagnostic yield and reclassification rate. In support of that position, multiple diagnostic studies and meta-analyses show that native T1 and T2 mapping add good sensitivity when it comes to detecting acute myocardial inflammation and diffuse injury compared with conventional T2-weighted imaging alone, and that the updated (2018) Lake Louise approach that incorporates mapping outperforms older criteria [30]. Moreover, some prospective early-CMR work that did include mapping (SMINC-2 [11]) reported a considerable increase in diagnostic yield (from 47% to 77%) when scans were performed earlier and mapping was also included, implying that mapping contributed to detecting transient oedema/inflammatory phenotypes that late non-mapping protocols miss. Mapping also reduces reader dependency for some non-ischemic diagnoses and can raise sensitivity and inter-reader reproducibility [10]. However, the fact that most included studies relied on conventional CMR protocols without mapping, while quantitative mapping was reported only in a limited number of specialised centres, can be interpreted in the context of heterogeneity in CMR protocols and healthcare infrastructure across the included studies. This has important diagnostic implications, as mapping techniques would increase sensitivity, particularly in patients without evident LGE pattern. Therefore, differences in reported diagnostic performance across studies may reflect the variability in technical capability rather than true differences in disease expression.

Secondly, reclassification rate is another important role that CMR plays in the diagnosis of MINOCA. The findings of this study [10] support the evidence that native mapping (T1 and T2) can achieve high diagnostic concordance with standard CMR (StdCMR) in many MINOCA cases, particularly in inflammatory pathologies where oedema (evaluated by T2 mapping) and diffuse injury (evaluated by T1 mapping) are prevalent. Furthermore, the study shows that mapping alone approaches the diagnostic accuracy of full contrast-enhanced CMR only when interpreted by an experienced reader. The markedly lower performance of non-experts, especially in myocardial infarction, highlights the value of LGE for identifying small focal infarcts that mapping often misses. The data suggest that mapping-driven protocols may safely shorten scan times and avoid contrast in selected patients, but mapping should not yet replace LGE when ischemic injury is suspected or when diagnostic confidence is low. These results reinforce the complementary roles of mapping and LGE and justify further work on workflow-optimised protocols, automated mapping quantification, and AI-assisted interpretation to reduce reader dependency.

Another important role of CMR is in analysing the prognosis of MINOCA and stratifying risk. The pathophysiology of MINOCA is heterogenous, and the prevalence rate of MACE is substantial [31]. The studies included showed that the presence and extent of LGE (number of segments, transmurality, and LGE%) helps identify patients at higher risk of MACE or rehospitalisation and that, when available, parametric mapping (T1 and T2) adds prognostic information beyond LGE alone (e.g., the study by Bergamaschi et al. [9] proved that both %LGE and early T2 mapping are independent predictors). This is evidence that a CMR protocol that includes LGE and mapping therefore increases diagnostic yield and allows for objective risk stratification, which can guide follow-up and secondary prevention. The relative heterogeneity in prognostic estimates could be explained by the fact that in some studies the timing of CMR was different (early vs. later), as late scans under-detect oedema of other transient findings [20], older cohorts do not have standardised mapping sequences, and some studies excluded non-ischemic entities. What can be clearly derived from the data is that early CMR (ideally within the first week) maximises the detection of transient pathology, including standardised mapping sequences as well as LGE gives better prognostic data, and using CMR guides secondary prevention and proper follow-up.

Another practical message from the studies included is that timing matters. The marked heterogeneity in imaging timing introduced an important risk of bias, since early CRM (1–2 weeks) was associated with higher diagnostic yield than examinations performed later (after two weeks). CMR is most sensitive when performed during early acute phase, shortly after patient presentation [14]. Therefore, the variability in imaging timing across the included studies and the differences in reported sensitivity may reflect the temporal evolution of the disease rather than true differences in CMR performance.

SMINC-2 [11] is a pragmatic demonstration that early CMR, performed within the first days after presentation, improves diagnostic yield in MINOCA. By changing the median scan time from 12 days (SMINC-1 [29]) to 3 days and incorporating advanced tissue characterisation (e.g., native T1 and ECV mapping), SMINC-2 increased the proportion of patients receiving a diagnosis from 47% to 77%, mainly through better detection of transient conditions (e.g., myocarditis and Takotsubo syndrome) that can normalise within weeks. Early timing captures transient oedema and wall-motion abnormalities, while mapping provides objective, quantitative detection of diffuse or subtle injury that conventional T2-weighted imaging may miss—a likely contributor to the marked rise in myocarditis detection (17% vs. 7%). Although the authors appropriately noted they could not fully disentangle the relative contribution of earlier imaging versus mapping/advanced sequences, the pattern of results favours timing as a dominant factor with mapping amplifying sensitivity for nonischaemic injury. SMINC-2 [11] therefore supports a clinical pathway of early CMR with standardised T1/T2/ECV mapping plus LGE for MINOCA triage: early scanning maximises the capture of transient pathology and mapping increases objectivity and sensitivity, together reducing indeterminate studies and improving diagnostic precision. SMINC-2 provides the strongest prospective, multicentre evidence to date that early mapping-enabled CMR should be central in the diagnosis of MINOCA.

Multimodality imaging could increase diagnostic clarity in MINOCA, as demonstrated by the studies conducted by Gerbaud et al. [27], Reynolds et al. [19], and Opolski et al. [28]. Reynolds et al.’s [19] multicentre study showed that combining CMR with OCT raised the diagnosis yield to 84%, and that mapping, when performed, meaningfully contributed to CMR interpretation; however, mapping was not available or interpretable in all patients included in the study, and OCT and CMR were not both performed in all patients, limiting concordance. Both the Gerbaud and Opolski studies showed that intracoronary imaging frequently reveals plaque disruption or thrombotic processes, even when coronary angiography appears normal. CMR helps confirm myocardial injury and localises the infarct-related territory using late gadolinium enhancement. The study by Gerbaud et al. also highlighted that, by combining both techniques, an underlying cause was identified in all patients included. However, until larger comparative studies are available, multimodality imaging (OCT + CMR) should be considered exploratory and reserved for cases in which they would impact management and treatment.

Another study [12] provided strong evidence that improving spatial resolution of LGE increases diagnostic yield in MINOCA by revealing small infarct areas and reducing equivocal studies. Importantly, most of the benefit reflected improved diagnostic confidence (clarifying uncertain transmural location) rather than only the discovery of completely new lesions. However, the study did not evaluate HR-LGE against modern parametric mapping: the authors stated that T1/T2 mapping techniques were not available, so no direct comparison or incremental analysis versus mapping/ECV can be drawn from their data. Consequently, HR-LGE should be viewed as a potentially powerful complement to, rather than a proven replacement for, standardised mapping sequences. A reasonable pathway for future research is a possible combined protocol (HR-LGE + T1/T2/ECV) to determine whether mapping adds sensitivity/specificity beyond HR-LGE and whether either method better predicts outcomes or changes management in MINOCA. That said, mapping is unlikely to fully substitute LGE in detecting very small focal infarcts, as LGE has better focal scar sensitivity, so an optimal strategy would be complementary: early CMR with additional mapping. [14]

Furthermore, the study by Masood et al. [15] showed that pre-angiography CMR altered the diagnosis and localisation in a substantial fraction of NSTEMI patients, particularly when angiography showed non-significant or multivessel disease. By localising an IRA in nearly half of angiography-negative cases and by revealing non-ischemic mimics in approximately 15% overall, CMR guided correct targeting of revascularisation, avoided unnecessary or inappropriate PCI (percutaneous coronary intervention), and changed medical therapy when a non-ischemic diagnosis (e.g., myocarditis and Takotsubo syndrome) was found. The practical consequence was improved triage: in stable patients who could safely undergo imaging before CAG, an early CMR-first pathway was appropriate. However, the study was single-centre and relatively small, and follow-up data on how CMR-guided revascularisation affected outcomes were not available. However, this study used LGE only, and parametric mapping (native T1/T2/ECV) was not performed, so some diffuse or very early inflammatory injuries that may alter triage could have been missed. In practice, the optimal strategy is complementary: early CMR with both LGE and standardised mapping would likely maximise the identification of ischemic and non-ischemic causes, further reduce the proportion of inconclusive studies, and strengthen decisions to perform invasive interventions.

The study by Macedo Conde et al. [13] reinforced a now-consistent message in MINOCA imaging: timing is pivotal. By showing markedly higher diagnostic rates when CMR was performed at ≤14 days (and a median scan interval of 12 days in patients with a diagnosis vs. 26 days in those without), they proved that many of the imaging features CMR detects (e.g., oedema, transient wall-motion abnormalities, and reversible myocardial injury) cannot be as easily identified if imaging is delayed, reducing the number of accurate diagnoses and potential mimickers, which could change management and avoid inappropriate therapies.

Lastly, where machine learning is concerned, some studies showed that AI could aid reclassification when LGE was available (radiomics) and potentially supported contrast-sparing protocol when LGE was not feasible. Di Noto et al. [6] demonstrated that radiomics of LGE could help distinguish ischemic from inflammatory enhancement and could reduce reader dependency in equivocal cases, which could be beneficial for MINOCA reclassification. Abdulkareem et al. [7] outlined some practical methods to predict post-contrast patterns from non-contrast imaging and showed the main technical hurdles (e.g., scar segmentation is still weak and classification accuracy remains modest), and they acknowledged that there is still room for improvement and further research is required before the use of AI can replace contrast enhanced imaging. All things considered, further studies are needed to demonstrate the possible benefits of AI and machine learning in the workup of MINOCA.

The integration of CMR in MINOCA is strongly shaped by differences in infrastructure and technical availability across different healthcare systems. Evidence from the recent literature indicated that CMR availability is often limited by scanner access, cost, and prolonged waiting times, with CMR services often concentrated in large centres and metropolitan areas [32]. These structural differences likely contribute to the heterogeneity observed across studies included in this review, particularly with regard to the timing of CMR, protocol completeness, diagnostic yield, and operator expertise. Emerging technologies, including artificial intelligence, have the potential to mitigate some of these issues; however, their adoption remains uneven. Consequently, differences in both infrastructure and AI implementation may partly explain inter-study variability and should be considered when interpreting the diagnostic performance and generalisability of CMR findings in MINOCA.

## 5. Limitations

This systematic review has several limitations that should be acknowledged. The significant heterogeneity across studies, particularly in CMR protocols and timing of imaging, affects comparability and may influence diagnostic yield. The predominance of conventional CMR techniques and the limited use of parametric mapping restrict the ability to draw firm conclusions about the added value of quantitative approaches. Additionally, most studies were observational and conducted in specialised centres, with protocols that cannot be uniformly implemented across different healthcare systems and geographic regions.

The findings of this review should be interpreted in the context of the inherent heterogenous nature of MINOCA, as shown in the recent literature [33]. MINOCA is not a single disease entity but rather a working diagnosis that includes a spectrum of mechanisms, each with distinct pathophysiological pathways and clinical implications. This diagnostic complexity also supports the development of advanced imaging techniques, including artificial intelligence and radiomics, which aim to refine tissue characterisation and improve diagnostic precision in this heterogenous population.

## 6. Conclusions

This systematic review confirms that CMR is a pivotal tool in MINOCA evaluation, as it contributes greatly to diagnostic yield, reclassification rate, and prognosis characterisation, as well as guiding correct timing of evaluation. When performed early and including comprehensive tissue characterisation (LGE plus T1/T2/ECV mapping), CMR can diagnose aetiology in most MINOCA patients—typically ≥70% in most studied cohorts. Mapping sequences in particular enhance detection of myocarditis and diffuse injury and provide quantitative prognostic markers. Early timing of imaging is critical: delaying CMR reduces sensitivity for transient findings. Importantly, CMR often reclassifies the initial MINOCA diagnosis, guiding more appropriate therapy. In the coming years, the integration of AI and other advanced techniques into CMR promises to further refine MINOCA imaging, improving accuracy and efficiency. Taken together, the current evidence shows that timely, mapping-inclusive CMR should be a standard component of MINOCA workup, with the potential for even greater insights as technology advances.

## Figures and Tables

**Table 1:** Key design features.

Author, Year	Design	Number of Patients	Mapping (T1/T2/ECV)	Timing (Median/Mean Days)	Primary Relevance	Key Finding
Bergamaschi 2024 [9]	Prospective multicentre cohort	198	Yes (native T1, T2 mapping)	4.8 ± 1.5 d	Mapping Prognosis Diagnostic yield	%LGE and T2 independent predictors of MACE
Gatti 2024 [10]	Multicentre retrospective	179	Yes (native T1 + T2 mapping in short protocol)	5 ± 4 d (mean)	Future directions (ShtCMR), Diagnostic yield	85% diagnostic agreement ShtCMR vs. StdCMR (experts).
Sörensson (SMINC-2) 2021 [11]	Prospective multicentre	148	Yes (native T1 + ECV)	3 d (mean)	Timing, Diagnostic yield	Diagnostic yield 77% (SMINC-2) vs. 47% (SMINC-1 historical).
Lintingre 2020 [12]	Prospective cohort	229 (HR-LGE in 172)	No mapping	4 d (mean)	Future directions (HR-LGE), Diagnostic yield	HR-LGE increased definite diagnoses 50% to 71% in HR subgroup; changed diagnosis in 45/172 (26%).
Macedo Conde 2024 [13]	Retrospective single-centre	163	No mapping	14 d (mean)	Timing, Diagnostic yield	Overall CMR diagnostic yield 121/163 (74.3%); scans ≤14 d had significantly higher yield.
Williams 2022 [14]	Prospective referral cohort	719	No mapping	30 d mean	Timing, Diagnostic yield	In subgroup (scan <14 d & peak hs-TnT ≥ 211 ng/L): diagnostic yield 94% (186/198).
Masood 2024 [15]	Prospective single-centre	70	No mapping	Pre-angiography	Reclassification, Diagnostic yield	CMR identified IRA in 67.1% (47/70) vs. CAG 61.4%; CMR produced new IRA in 23% (16/70).
Camastra 2017 [16]	Retrospective cohort	190	No mapping	4 ± 2 d (mean)	Timing, Diagnostic yield	CMR diagnostic yield 85%
Bucciarelli 2023 [17]	Retrospective single-centre	135	No mapping	28 d (mean)	Prognosis, Reclassification	LGE pattern led to therapy change in 22% (30/135); transmural LGE predicted worse outcomes.
Heidecker 2019 [18]	Retrospective single-centre	556	No mapping	10 d (mean)	Reclassification, Diagnostic yield	Systematic CMR increased myocarditis detection 4.9-fold
Reynolds (WOMEN-HARP) 2021 [19]	Prospective multicentre (women with MI & <50% stenosis)	301 enrolled; 170 MINOCA	Partial (T1 mapping interpretable in ~65% of CMR subset)	6 d (mean)	Reclassification, Multimodality (OCT + CMR), diagnostic yield	OCT identified culprit in 46.2% (67/145); CMR abnormal in 74.1% (86/116); OCT + CMR combined diagnosis in 84.5% (98/116).
Di Notto 2019 [6]	Retrospective single-centre	173	No mapping	Not consistently reported	Future directions	Radiomics applied to LGE regions can discriminate infarction from inflammation
Abdulkareem 2022 [7]	Retrospective single-centre	272	No mapping	Not consistently reported	Future directions	Predict post-contrast information from pre-contrast cine images
Dastidar 2019 [20]	Retrospective registry	388	No mapping	37 d (median)	Prognostic Diagnostic yield, timing	Increased mortality rates (4.5% over a median of 3.5 years) in MI patients; highest mortality in cardiomyopathy/Takotsubo group (15% vs. 4.5% MI vs. 2% myocarditis/normal
Ananthakrishna 2022 [21]	Prospective cohort	229	No mapping	6 d (median)	Prognostic, Diagnostic yield, Timing	24% experienced MACE long-term (median follow-up 7.1 years)
Vicente-Ibarra 2021 [22]	Retrospective registry	120	No mapping	12 d	Prognostic, diagnostic yield	follow-up median 2.9 years. MACE in 35.8%; infarct size/number of LGE segments strongly associated with worse prognosis, involvement of ≥3 segments nearly tripled MACE risk
Biere 2017 [23]	Retrospective cohort	131	No mapping	Not consistently reported	Prognostic, diagnostic yield	Ventricular arrhythmias occurred in ~13.8% during hospitalisation; LGE transmural extent and ST-elevation independently predicted ventricular arrhythmic events. No sudden cardiac deaths recorded at 1 year
Panovsky 2017 [24]	Retrospective multicentre cohort	136	No mapping	7.4 (mean)	Diagnostic yield, timing	Final diagnosis in 57% of patients
Pathik 2015 [25]	Prospective cohort	125	No mapping	6 d (mean)	Reclassification rate, Diagnostic yield	Final diagnosis in 87% of patients
Samaras 2025 [26]	Retrospective single centre cohort	1596	No mapping	Not consistently reported	Prognostic, Diagnostic yield	CMR clarified aetiology in >70% of MINOCA/mimicker patients; long-term all-cause mortality in MINOCA comparable to MI-CAD (32.1% vs. 30.9%)
Gerbaud 2020 [27]	Prospective two-centre study	40	No mapping	3 d (mean)	Diagnostic yield, future directions (HR-LGE)	CMR identified myocardial infarction in 31/40 patients; Combined OCT-CMR imaging provided a definitive mechanism or diagnosis in 100% of patients
Opolski 2019 [28]	Prospective cohort	38	No mapping	4 d (mean)	Diagnostic yield, future directions	CMR was used to identify infarct-related artery, enabling correlation between coronary plaque morphology and myocardial injury

## Data Availability

All data used in this systematic review are from previously published studies, which are cited and available within the article and its references. No new data were generated.
